# A comprehensive study evaluating germline *FANCG* variants in predisposition to breast and ovarian cancer

**DOI:** 10.1002/cam4.70103

**Published:** 2024-08-16

**Authors:** Jana Soukupova, Barbora Stastna, Madiha Kanwal, Jan Hojny, Petra Zemankova, Marianna Borecka, Leona Cerna, Marta Cerna, Monika Cerna, Vaclava Curtisova, Tatana Dolezalova, Petra Duskova, Lenka Foretova, Ondrej Havranek, Klara Horackova, Milena Hovhannisyan, Lucie Hruskova, Stepan Chvojka, Marketa Janatova, Maria Janikova, Sandra Jelinkova, Pavel Just, Marta Kalousova, Petra Kleiblova, Marcela Kosarova, Monika Koudova, Jan Kral, Michaela Krausova, Vera Krutilkova, Eva Machackova, Katerina Matejkova, Renata Michalovska, Petr Nehasil, Barbora Nemcova, Jan Novotny, Matous Palek, Pavel Pesek, Marketa Safarikova, Ondrej Scheinost, Drahomira Springer, Lenka Stolarova, Viktor Stranecky, Ivan Subrt, Spiros Tavandzis, Eva Tureckova, Kamila Vesela, Zdenka Vlckova, Michal Vocka, Tomas Zima, Libor Macurek, Zdenek Kleibl, the CZECANCA consortium

**Affiliations:** ^1^ Institute of Medical Biochemistry and Laboratory Diagnostics, First Faculty of Medicine, Charles University and General University Hospital in Prague Prague Czech Republic; ^2^ Laboratory of Cancer Cell Biology Institute of Molecular Genetics of the Czech Academy of Sciences Prague Czech Republic; ^3^ Department of Biochemistry, Faculty of Science Charles University Prague Czech Republic; ^4^ Institute of Pathology, First Faculty of Medicine, Charles University and General University Hospital in Prague Prague Czech Republic; ^5^ Institute of Pathological Physiology, First Faculty of Medicine, Charles University Prague Czech Republic; ^6^ Centre for Medical Genetics and Reproductive Medicine, GENNET Prague Czech Republic; ^7^ Institute of Medical Genetics, University Hospital Pilsen Pilsen Czech Republic; ^8^ Department of Medical Genetics Faculty of Medicine and Dentistry, University Hospital Olomouc, Palacky University Olomouc Czech Republic; ^9^ Hospital Ceske Budejovice Ceske Budejovice Czech Republic; ^10^ Department of Cancer Epidemiology and Genetics Masaryk Memorial Cancer Institute Brno Czech Republic; ^11^ Institute of Biology and Medical Genetics, First Faculty of Medicine, Charles University and General University Hospital in Prague Prague Czech Republic; ^12^ BIOCEV, First Faculty of Medicine Charles University Vestec Czech Republic; ^13^ Department of Medical Genetics GHC Genetics Prague Czech Republic; ^14^ Department of Medical Genetics Pronatal Prague Czech Republic; ^15^ Department of Medical Genetics, AGEL Laboratories AGEL Research and Training Institute Novy Jicin Czech Republic; ^16^ Department of Genetics and Microbiology, Faculty of Science Charles University in Prague Prague Czech Republic; ^17^ Department of Paediatrics and Inherited Metabolic Disorders, First Faculty of Medicine Charles University and General University Hospital in Prague Prague Czech Republic; ^18^ Institute for Clinical and Experimental Medicine Prague Czech Republic; ^19^ Department of Oncology, First Faculty of Medicine Charles University and General University Hospital in Prague Prague Czech Republic

**Keywords:** breast cancer, Fanconi anemia complementation group G, functional analysis, germline genetic testing, hereditary tumors, ovarian cancer

## Abstract

**Background:**

Monoallelic germline pathogenic variants (GPVs) in five Fanconi anemia (FA) genes (*BRCA1/FANCS*, *BRCA2/FANCD1*, *PALB2/FANCN*, *BRIP1/FANCJ*, and *RAD51C/FANCO*) confer an increased risk of breast (BC) and/or ovarian (OC) cancer, but the role of GPVs in 17 other FA genes remains unclear.

**Methods:**

Here, we investigated the association of germline variants in *FANCG/XRCC9* with BC and OC risk.

**Results:**

The frequency of truncating GPVs in *FANCG* did not differ between BC (20/10,204; 0.20%) and OC (8/2966; 0.27%) patients compared to controls (6/3250; 0.18%). In addition, only one out of five tumor samples showed loss‐of‐heterozygosity of the wild‐type *FANCG* allele. Finally, none of the nine functionally tested rare recurrent missense *FANCG* variants impaired DNA repair activities (FANCD2 monoubiquitination and FANCD2 foci formation) upon DNA damage, in contrast to all tested *FANCG* truncations.

**Conclusion:**

Our study suggests that heterozygous germline *FANCG* variants are unlikely to contribute to the development of BC or OC.

## Graphical abstract

*FANCG*, together with established breast/ovarian predisposition genes *BRCA1* and *BRCA2* belongs to the Fanconi anemia gene family; however, little is known about cancer predisposition in carriers of *FANCG* germline alterations. Here we demonstrate that these individuals does not have increased breast and ovarian cancer risk, because the frequency of *FANCG* pathogenic variants among cancer patients are not different from that in controls, the tumors in *FANCG* carriers showed no loss‐of‐heterozygozity of wild type allele, and functional in vitro analysis found that any tested rare missense variant impaired FANCG capacity in DNA repair.

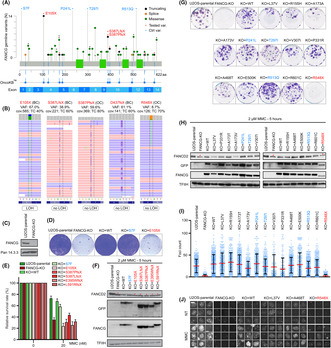

## 1 BACKGROUND

The Fanconi anemia (FA) genes encode at least 22 proteins that form multiprotein complexes involved in the resolution of interstrand DNA interstrand cross‐links and the precise repair of DNA double‐strand breaks via homologous recombination.^1^ While biallelic germline pathogenic variants (GPVs) in these genes cause FA (a syndrome characterized by morphologic abnormalities, bone marrow failure, and increased risk of malignancy development), monoallelic GPVs in five FA genes (including *BRCA1/FANCS*, *BRCA2/FANCD1*, *PALB2/FANCN*, *BRIP1/FANCJ*, *RAD51C/FANCO*) confer an increased risk of breast (BC) and/or ovarian (OC) cancer. The role of monoallelic GPVs in other FA genes in BC/OC predisposition remains unclear.^2^, ^3^

*FANCG/XRCC9* encodes a protein of the FA core complex. The primary role of the FA core complex is to monoubiquitinate FANCD2, leading to FANCD2‐FANCI heterodimerization (formation of ID2 complex) and the subsequent activation of downstream DNA repair effectors.^4^ Notably, within the core complex, FANCG interacts with BRCA1 and BRCA2, the proteins encoded by two major hereditary BC/OC predisposition genes.^4^

Biallelic GPVs in *FANCG* cause FA complementation group G (FA‐G; OMIM#614082). *FANCG*, with *FANCA* and *FANCC*, belongs to the most frequently mutated genes responsible for approximately 80% of FA patients worldwide.^5^ Heterozygous *FANCG* GPVs have been identified episodically in cancer patients and their association with cancer risk remains uncertain.^6^ Carriers have been described in patients with BC,^7^, ^8^ OC,^9^ and pancreatic^10^, ^11^, ^12^ cancers, a tumor spectrum characteristic for carriers of GPVs in established FA cancer predisposition genes (including *BRCA1*, *BRCA2*). Moreover, our previous study identified 5/1333 (0.38%) *FANCG* GPVs in OC patients (included in this dataset), suggesting a possible association of *FANCG* GPVs with OC.^13^ To clarify the role of *FANCG* GPVs in BC/OC predisposition, we performed a case–control analysis and the functional in vitro testing of selected germline *FANCG* variants.

## 2 MATERIALS AND METHODS

The frequencies of GPVs (truncating/spliceogenic) and rare missense *FANCG* variants (Table 1) were retrieved from the CZECANCA (CZEch CANcer panel for Clinical Application) database version 6 (May 20, 2023), a collection of anonymized phenotype/genotype data from the Czech national consortium (www.czecanca.cz) for germline genetic testing, as we described previously.^14^ The datasets included 10,204 female BC patients (including 6753 patients who met national germline genetic testing criteria based on NCCN guidelines^15^ and 3451 patients who did not meet testing criteria but were analyzed identically), 2966 unselected OC patients (1333 from previous study and 1633 newly added; all indicated for germline genetic testing in the Czech Republic), and 3250 female population‐matched controls (adult volunteers who did not meet germline genetic testing criteria). All individuals provided written informed consent with genetic testing approved by the Ethics Committee of the First Faculty of Medicine and General University Hospital in Prague and were Czechs of Central European origin. A burden case–control analysis was performed to determine the risk in *FANCG* GPVs carriers.

**TABLE 1:** Identified germline *FANCG* variants.

Exon	TPR	Variant description (NM_004629.2)		ClinVar		InterVar	Breast ca. (*N* = 10,204)	Ovarian ca. (*N* = 2966)	CTRLs (*N* = 3250)
(i)	#	c.	p.	Variant ID	Review status	Class	*N* (%)	*N* (%)	*N* (%)
*Truncating variants*									
i1		85‐1G>C	?^b^	n.a.	n.a.	n.a.	–	–	1 (0.03)
4		313G>T	Glu105Ter^a^, ^b^	6712	5**‐‐	P	9 (0.09)	2 (0.07)	3 (0.09)
4		373_374del	Val125ProfsTer29	n.a.	n.a.	n.a.	1 (0.01)	–	–
5		520del	Ser174LeufsTer8	n.a.	n.a.	n.a.	–	1 (0.03)	–
5		522_523del	Lys175GlyfsTer14	n.a.	n.a.	n.a.	–	1 (0.03)	–
5		560delC	Pro187GlnfsTer5^b^	1452560	5*‐‐‐	n.a.	1 (0.01)	–	–
10		1158delC	Ser387ProfsTer16^a^	619961	5/4**‐‐	n.a.	–	1 (0.03)	–
10		1158dupC	Ser387LeufsTer9^a^	623182	5/4**‐‐	n.a.	5 (0.05)	1 (0.03)	–
10		1183_1192del	Glu395TrpfsTer5^a^, ^b^	41224	‐‐‐‐	n.a.	2 (0.02)	–	1 (0.03)
10		1309_1310dup	Asp437GlufsTer82	1073453	5**‐‐	n.a.	2 (0.02)	–	–
i11		1480+1G>T	?	n.a.	n.a.	n.a.	–	–	1 (0.03)
13		1642C>T	Arg548Ter^a^, ^b^	574728	5**‐‐	P	–	1 (0.03)	–
14		1772del	Leu591ArgfsTer3^a^, ^b^	2136764	4/3*‐‐‐	n.a.	–	1 (0.03)	–
All truncating variants							20 (0.20)	8 (0.27)	6 (0.18)
odds ratio (95% confidence interval); *p*‐value							1.1 (0.4–2.7); 0.9	1.5 (0.5–4.2); 0.5	Ref.
*Rare missense variants*									
2		109C>G	Leu37Val^a^	1319087	3*‐‐‐	LB	4 (0.04)	–	
2		122A>G	Gln41Arg	n.a.	n.a.	LB	2 (0.02)	–	1 (0.03)
4		338G>A	Arg113Lys	526405	3*‐‐‐	LB		–	3 (0.09)
4		401A>C	Glu134Ala	n.a.	n.a.	VUS	1 (0.01)	–	–
4		418C>T	His140Tyr	n.a.	n.a.	VUS	1 (0.01)	–	–
4		421C>T	Arg141Cys	239967	3**‐‐	VUS		1 (0.03)	–
4		422G>A	Arg141His	999134	3**‐‐	LB	2 (0.02)	–	–
4		464G>A	Arg155His^a^	456236	3**‐‐	LB	2 (0.02)	2 (0.07)	–
4		486A>T	Leu162Phe	1416142	3**‐‐	LB	1 (0.01)	–	1 (0.03)
5		517G>A	Ala173Thr^a^	2085419	3*‐‐‐	LB	1 (0.01)	–	2 (0.06)
5		518C>T	Ala173Val^a^	n.a.	n.a.	VUS	–	–	3 (0.09)
5		580C>G	Pro194Ala	n.a.	n.a.	VUS	1 (0.01)	–	–
6		724C>T	Arg242Trp	912780	3**‐‐	VUS	–	–	1 (0.03)
6	1	761C>T	Ser254Phe	1319083	3*‐‐‐	VUS	2 (0.02)	–	1 (0.03)
7	1	794C>T	Ala265Val	912779	3**‐‐	VUS	–	–	1 (0.03)
7	1	835T>G	Trp279Gly	1336044	3*‐‐‐	VUS	1 (0.01)	1 (0.03)	–
7		910G>C	Glu304Gln	2577889	3*‐‐‐	VUS	1 (0.01)	–	–
7		917T>C	Leu306Pro	2202538	3*‐‐‐	VUS	1 (0.01)	–	–
7		919G>A	Val307Ile^a^	n.a.	n.a.	VUS	3 (0.03)	–	–
8		956C>T	Pro319Leu	n.a.	n.a.	LB	1 (0.01)	–	2 (0.06)
8		992C>T	Pro331Leu	n.a.	n.a.	LB	1 (0.01)	–	–
8		992C>G	Pro331Arg^a^	802483	3**‐‐	LB	6 (0.06)	–	3 (0.09)
8		1027C>G	Gln343Glu	914731	3*‐‐‐	VUS	1 (0.01)	–	–
8	2	1070C>T	Thr357Met	1806248	3**‐‐	LB	2 (0.02)	–	–
8	2	1076G>A	Arg359Lys	1199306	3*‐‐‐	VUS	2 (0.02)	–	–
9		1143G>C	Arg381Ser	n.a.	n.a.	VUS	1 (0.01)	–	1 (0.03)
10		1157C>A	Pro386His	914728	3**‐‐	LB	–	2 (0.07)	–
10		1268G>A	Arg423His	456228	3**‐‐	VUS	2 (0.02)	2 (0.07)	–
10		1298G>A	Arg433Gln	644471	3*‐‐‐	LB	–	1 (0.03)	–
10		1328A>G	Lys443Arg	n.a.	n.a.	LB	1 (0.01)	–	–
10	3	1367A>T	His456Leu	579904	3**‐‐	VUS	2 (0.02)	1 (0.03)	–
10	3	1402G>A	Ala468Thr^a^	n.a.	n.a.	VUS	1 (0.01)	1 (0.03)	1 (0.03)
12		1492A>C	Asn498His	836334	3**‐‐	LB	–	1 (0.03)	–
12		1498G>A	Glu500Lys^a^	2056153	3*‐‐‐	VUS	5 (0.05)	–	2 (0.06)
12		1505G>T	Gly502Val	1721737	3*‐‐‐	VUS	2 (0.02)	–	–
12	4	1546G>A	Ala516Thr	2043453	3*‐‐‐	VUS	2 (0.02)	–	–
12	4	1558C>T	Arg520Cys	n.a.	n.a.	VUS	–	1 (0.03)	–
12	4	1586A>G	Gln529Arg	2038189	3*‐‐‐	VUS	1 (0.01)	–	–
13		1643G>A	Arg548Gln	1692835	3*‐‐‐	LB	1 (0.01)	1 (0.03)	–
13		1685A>G	Asp562Gly	n.a.	n.a.	VUS	–	1 (0.03)	–
13		1688G>A	Arg563Gln	846051	3**‐‐	VUS	1 (0.01)	–	–
13		1754C>G	Ala585Gly	n.a.	n.a.	VUS	1 (0.01)	–	–
14		1801C>T	Arg601Cys^a^	666016	3*‐‐‐	VUS	3 (0.03)	1 (0.03)	–
14		1802G>A	Arg601His	n.a.	n.a.	LB	–	–	2 (0.06)
All rare missense variants							59 (0.58)	16 (0.54)	24 (0.74)
*Frequent missense variants*									
1		20C>T	Ser7Phe^a^	134358	1/2/3*‐‐‐	LB	30 (0.30)	9 (0.30)	12 (0.37)
6		722C>T	Pro241Leu^a^	221037	2/3*‐‐‐	VUS	10 (0.10)	–	1 (0.03)
7		890C>T	Thr297Ile^a^	134367	1/2**‐‐	LB	27 (0.26)	8 (0.27)	9 (0.27)
12		1538G>A	Arg513Gln^a^	134361	1/2/3*‐‐‐	LB	112 (1.10)	41 (1.38)	42 (1.29)
All frequent missense variants							179 (1.75)	58 (1.96)	64 (1.97)

*Note*: InterVar (https://wintervar.wglab.org/). ClinVar review status was 2023/12/01 and express ClinVar numerical classification (class 1–5 when available) and aggregate ClinVar review status (zero to four stars: ‐‐‐‐ to ****).

Abbreviations: c., cDNA; (i), intron; LB, likely benign; n.a., not available; P, pathogenic; p., protein; TPR, tetratricopeptide repeat (numbers 1–4); VUS, variant of unknown significance.

^a^ Variant analyzed by functional assays in this study.

^b^ Variant described in FA patient.

**FIGURE 1:**
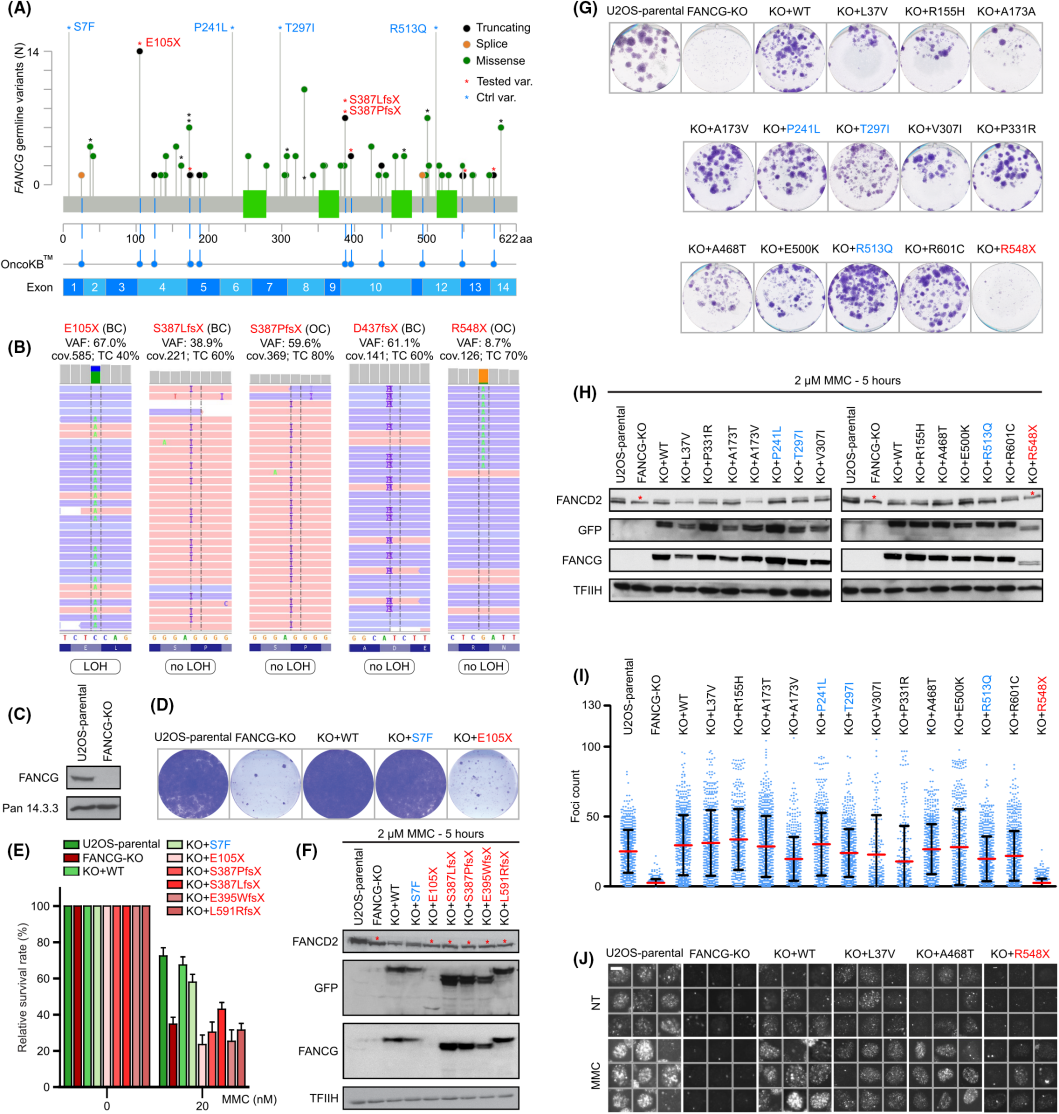
(A) Distribution of germline *FANCG* variants identified in patients and controls (created using https://www.cbioportal.org/). Asterisks indicate variants included in functional testing (blue—missense variants with minor allele frequency (MAF) >0.002 in gnomAD database that were selected as fully‐functional controls; red—truncations). All truncations and splicing alterations were considered pathogenic by OncoKB (www.oncokb.org). Exon structure corresponds to NM_004629.2 reference. (B) DNA sequencing from breast (BC) and ovarian (OC) cancer FFPE samples available from five patients carrying truncating *FANCG* variants. (C–J) Functional characterization of DNA damage response in FANCG variants expressed in U2OS‐FANCG‐KO cells lacking endogenous FANCG. (C) Immunoblot showing the level of endogenous FANCG in U2OS‐parental and U2OS‐FANCG‐KO cells (Santa Cruz, sc‐393,382). As loading control was used protein Pan 14‐3‐3 (Santa Cruz, sc‐133,233). (D) Colony formation assay of parental U2OS, U2OS‐FANCG‐KO (FANCG‐KO), and FANCG‐KO stably transfected with truncated FANCG (red text) or fully‐functional FANCG‐S7F missense variant (blue text) after treatment with 1 nM MMC for 14 days. Colonies were fixed with ethanol (70% v/v) and stained with crystal violet. Note that FANCG‐KO cells and FANCG‐KO cells expressing the most frequent truncating variant p.E105X fail to grow in MMC. (E) Survival assay of parental U2OS cells, FANCG‐KO cells, and FANCG‐KO stably transfected with FANCG variants demonstrates that all analyzed truncating variants fail to rescue survival following MMC treatment. Relative cell proliferation was determined by resazurin assay (*n* = 3; mean with SD displayed). (F) Parental U2OS, FANCG‐KO cells, and FANCG‐KO cells stably transfected with FANCG variants were treated with MMC (2 μM, 5 h) and analyzed by immunoblotting with FANCD2 antibody (Abcam, ab108928) to visualize FANCD2 monoubiquitination. A red asterisk indicates the lack of FANCD2 monoubiquitination in FANCG‐KO cells and in all cells expressing analyzed truncating variants. Immunoblotting for GFP (Roche, 11,814,460,001), FANCG (Santa Cruz, sc‐393,382), and transcription factor TFIIH (sc‐293; Santa Cruz) were used as loading controls. (G) A colony formation assay indicates that all tested missense variants rescued cell growth following MMC treatment (1 nM, 7 days). (H) Immunoblotting performed as in 1F demonstrated rescue of FANCD2 monoubiquitination in FANCD‐KO cells expressing all analyzed missense variants in contrast to its loss in FANCG‐KO controls and FANCG‐KO cells expressing the C‐terminal truncating variant p.R548X. (I) Quantitative analysis of FANCD2 nuclear foci formation. U2OS, FANCG‐KO, and reconstituted FANCG‐KO stables cell lines were treated with 2 μM MMC for 5 h, pre‐extracted, fixed and stained with DAPI and FANCD2 antibody (Abcam, ab108928) and imaged using Olympus ScanR microscope equipped with 60×/1.42 OIL objective. The number of nuclear FANCD2 foci was determined using spot detection module in ScanR analysis software. Each dot represents one cell, red bar indicates mean, and bars are SDs. Representative out of two independent experiments. Note that FANCD2 foci do not form in FANCG‐KO cells and all tested missense variants rescued FANCD2 foci formation. FANCG‐KO expressing p.R548X truncation (red text) served as negative control (at least 270 cells were analyzed per condition). (J) Representative microscopy images from (I) showing FANCD2 foci formation in nuclei stained with DAPI after 2 μM MMC treatment (5 h) in U2OS cells, reduced foci formation in FANCG‐KO cells and FANCG‐KO cells expressing p.R548X and rescued FANCD2 foci formation in FANCG‐KO cells expressing wild‐type FANCG and all missense variants (scale bars 10 μm). VAF, variant allele frequency; cov., coverage; TC, percentage of tumor cells in sequenced sample; LOH/no LOH, presence/absence of loss of heterozygosity.

The selected germline *FANCG* variants were functionally evaluated in vitro. Briefly, endogenous *FANCG* was knocked out in U2OS cells (FANCG‐KO) by CRISPR/Cas9 technology using the pX458 plasmid (Addgene #48138) expressing sgRNAs targeting exons 1 and 4. pEGFP‐C1‐FANCG‐T2A‐Puro plasmids for the expression of wild‐type FANCG or its individual variants were generated by Gibson assembly and were stably transfected into FANCG‐KO cells. FANCG variants were tested by treating the reconstituted cells with mitomycin (MMC) followed by evaluation of FANCD2 monoubiquitination by immunoblotting, localization of FANCD2 in nuclear DNA repair foci by high content ScanR microscopy, and by colony formation and survival assay (Figure 1C–J, details available upon request). Four recurrent missense variants (Table 1; MAF_gnomAD_ >0.002) and wild‐type FANCG were considered positive, fully functional controls, while truncating GPVs and FANCG‐KO cells served as negative, functionally dead controls.

Loss‐of‐heterozygosity (LOH) at the *FANCG* locus was evaluated in five *FANCG* GPV carriers using in‐house 359 genes NGS panel, analyzing DNA from formalin‐fixed paraffin‐embedded (FFPE) tumor samples. LOH was detected using the Copy Number Variant Detection module (CLC Genomics Workbench v23.0.5) along with the frequency of detected mutations of germline origin, considering the percentage of tumor fraction in the analyzed FFPE sample.

## 3 RESULTS

We identified 57 different, rare heterozygous, germline *FANCG* variants (Figure 1A; Table 1) including 13 frameshift, stop‐gain, or spliceogenic variants considered as GPVs (localized before the most N‐terminal GPV identified in FA‐G patients, c.1795_1804del—ClinVar ID: 6718). However, the frequency of GPVs in patients with BC (20/10,204; 0.20%) or OC (8/2966; 0.27%) did not differ from that in controls (6/3250; 0.18%; Table 1). The frequency of *FANCG* GPV carriers in BC was insignificantly higher among patients who were not indicated for germline genetic testing than among those who were indicated [10/3451 (0.29%) vs. 10/6753 (0.15%); *p* = 0.19]. We found no evidence for the association of *FANCG* GPVs and ER‐negative BC identified in 4/17 (23.5%) carriers and 2321/8383 (27.7%) all BC patients with known ER status, suggested by Nierenberg et al. recently.^8^ In addition, the analysis of five available tumor samples from patients with *FANCG* GPVs revealed only one case of LOH at the *FANCG* locus (Figure 1B).

To test the pathogenicity of *FANCG* missense variants, we expressed selected variants in U2OS FANCG‐KO cells and evaluated their overall DNA repair capacity following genotoxic treatment. Specifically, we assessed FANCD2 monoubiquitination and its localization to nuclear foci as a readout of the FANCG‐dependent FA core complex functionality. Our assays confirmed a clear defect in the overall DNA repair and FA core complex activity in FANCG truncations (Figure 1D–F) but we found no evidence of functional impairment in any of the FANCG missense variants tested (Figure 1G–J).

## 4 DISCUSSION

Although heterozygous GPVs in five FA genes (*BRCA1*, *BRCA2*, *PALB2*, *BRIP1*, or *RAD51C*) confer high/moderate BC/OC risk, we found no association between *FANCG* GPVs and BC/OC risk. Regarding the case–control evidence for OC predisposition, our results are in agreement with a previous study by Song et al. who also found no association between GPVs in other FA genes (including *FANCG*) and OC risk. Specifically, Song's et al. identified 11/6184 (0.17%) *FANCG* GPVs carriers in OC patients compared to 8/6089 (0.13%) such carriers in controls (OR = 1.4; 95% CI 0.5–3.4).^9^ In addition, we detected LOH, an important marker of allelic imbalance indicating the presence of a driver mutation in a tumor suppressor gene, in only one of the five tumors analyzed. Finally, our in vitro functional assays showed that all rare missense variants analyzed did not affect the role of FANCG in DNA repair. Taken together, our study strongly suggests that heterozygous germline *FANCG* variants (including GPVs) do not confer an increased risk of BC or OC.

## AUTHOR CONTRIBUTIONS

**Jana Soukupova:** Conceptualization (lead); data curation (lead); formal analysis (lead); funding acquisition (equal); investigation (lead); methodology (equal); project administration (equal); supervision (equal); writing – original draft (equal); writing – review and editing (equal). **Barbora Stastna:** Investigation (equal); methodology (equal); visualization (equal); writing – review and editing (supporting). **Madiha Kanwal:** Investigation (equal); methodology (equal); visualization (equal). **Jan Hojny:** Investigation (equal); methodology (equal); writing – original draft (supporting); writing – review and editing (supporting). **Petra Zemankova:** Software (lead); writing – original draft (supporting); writing – review and editing (supporting). **Marianna Borecka:** Data curation (equal). **Leona Cerna:** Data curation (equal). **Marta Cerna:** Data curation (equal). **Monika Cerna:** Data curation (equal). **Vaclava Curtisova:** Data curation (equal). **Tatana Dolezalova:** Investigation (equal). **Petra Duskova:** Data curation (equal). **Lenka Foretova:** Data curation (equal). **Ondrej Havranek:** Data curation (equal); writing – review and editing (equal). **Klara Horackova:** Data curation (equal). **Milena Hovhannisyan:** Data curation (equal). **Lucie Hruskova:** Data curation (equal). **Stepan Chvojka:** Data curation (equal). **Marketa Janatova:** Data curation (equal); formal analysis (equal). **Maria Janikova:** Data curation (equal). **Sandra Jelinkova:** Data curation (equal). **Pavel Just:** Investigation (equal). **Marta Kalousova:** Resources (equal). **Petra Kleiblova:** Data curation (equal). **Marcela Kosarova:** Data curation (equal). **Monika Koudova:** Data curation (equal). **Jan Kral:** Data curation (equal). **Michaela Krausova:** Investigation (equal). **Vera Krutilkova:** Data curation (equal). **Eva Machackova:** Data curation (equal). **Katerina Matejkova:** Software (equal). **Renata Michalovska:** Data curation (equal). **Petr Nehasil:** Software (equal). **Barbora Nemcova:** Investigation (equal). **Jan Novotny:** Data curation (equal). **Matous Palek:** Methodology (equal). **Pavel Pesek:** Formal analysis (equal). **Marketa Safarikova:** Resources (equal). **Ondrej Scheinost:** Data curation (equal). **Drahomira Springer:** Data curation (equal). **Lenka Stolarova:** Methodology (equal). **Viktor Stranecky:** Software (equal). **Ivan Subrt:** Data curation (equal). **Spiros Tavandzis:** Data curation (equal). **Eva Tureckova:** Investigation (equal). **Kamila Vesela:** Data curation (equal). **Zdenka Vlckova:** Data curation (equal). **Michal Vocka:** Data curation (equal). **Tomas Zima:** Funding acquisition (supporting); resources (equal). **Libor Macurek:** Conceptualization (equal); investigation (equal); methodology (lead); supervision (equal); visualization (lead); writing – original draft (equal); writing – review and editing (equal). **Zdenek Kleibl:** Conceptualization (equal); data curation (equal); funding acquisition (lead); investigation (lead); supervision (equal); visualization (equal); writing – original draft (lead); writing – review and editing (lead).

## CONFLICT OF INTEREST STATEMENT

The authors declare no conflict of interest.

## ETHICS STATEMENT

All individuals provided written informed consent with genetic testing approved by the Ethics Committee of the First Faculty of Medicine and General University Hospital in Prague and the study was conducted in accordance with the Declaration of Helsinki.

## Data Availability

Research data supporting this publication are provided in this article. Details of the methods are available from the corresponding author upon reasonable request.
